# Development of a Brief Assessment Tool to Evaluate Early Low Nutrition Risk in Community Elderly: Creation of the Tool and Examination of Its Reliability and Criterion-related Validity

**DOI:** 10.2188/jea.JE20240056

**Published:** 2025-02-05

**Authors:** Shoji Shinkai, Miki Narita, Hiroshi Murayama, Akihiko Kitamura, Yoshinori Fujiwara

**Affiliations:** 1 Kagawa Nutrition University, Saitama, Japan; 2 Tokyo Metropolitan Institute of Gerontology, Tokyo, Japan; 3 Tokyo Online University, Tokyo, Japan; 4 Yao City Public Health Center, Osaka, Japan

**Keywords:** undernutrition, risk assessment tool, community elderly

## Abstract

**Background:**

To date, a simple assessment tool to evaluate early low nutrition risk in general older population has not been available. This study aimed to create such a tool and examined its reliability and criterion-related validity.

**Methods:**

1,192 community elderly with a mean age of 74.7 (standard deviation, 5.8) years responded to a questionnaire consisting of 48 (Hatoyama) or 34 items (Kusatsu), which have been reported to be associated with nutritional state in older people. Item analysis was conducted on the 34 common items, and items were selected based on the following criteria: adequate pass rates and discriminative power, no gender and regional differences, and a certain level of commonality based on factor analysis. Next, the factor structure of the candidate items was examined through exploratory factor analysis, and confirmatory factor analysis was conducted as the final scale structure. Furthermore, Spearman’s partial rank correlation coefficients (sex- and age-adjusted) between the created index and important health indicators were examined to determine the criterion-related validity.

**Results:**

Finally, we obtained a semantic coherence of four factors (named health beliefs, dietary status, physical activity, and food-related quality of life) totaling 13 items; confirmatory factor analysis of the four-factor solution yielded good model fit values, χ^2^ (59) = 275.4 (*P* < 0.001), comparative fit index = 0.930, and root mean square error of approximation = 0.056. The factor loadings for each factor ranged from 0.43 to 0.82, indicating adequate loadings. The reliability of the index was shown to be high by Good-Poor analysis and Cronbach’s α. The index showed statistically significant correlations with all health indicators.

**Conclusion:**

We have developed a simple assessment tool to evaluate early low nutrition risk in the general older population.

## INTRODUCTION

Low nutrition, which tends to occur in old age, is one of the important public health issues worldwide.^1^ The second phase of the Health Japan 21, the direction of health policy issued by the Ministry of Health, Labour and Welfare of Japan, also noted the prevention of undernutrition as a health issue for older people and calls for effective countermeasures to address this issue.^2^ In general, malnutrition among older adults in the community is often mild, but it becomes more serious with advancing functional decline due to the effects of chronic diseases and aging.^3^ According to the National Health and Nutrition Examination Survey Japan in 2019, the frequency of body mass index (BMI) below 20 kg/m^2^ among the general older population aged 65 years and older is 12.4% for men and 20.7% for women, but the frequency increases with age, reaching 17.2% and 27.9% in those aged 85 years and older.^4^ On the other hand, previous studies often use a serum albumin level of less than 4.0 g/dL as an indicator of low nutrition.^5^ According to this, low nutrition is found in 20–40% of elderly patients requiring nursing care and in 30–50% of hospitalized elderly patients.^6^

Using a dataset from the 2003–2011 National Health and Nutrition Examination Survey Japan, Yokoyama et al^7^ reported that the intake of various foods and nutrients declines with increasing age after the age of 65 years, suggesting that the risk of undernutrition has already emerged in older persons, even if they are independent in their daily livings. Therefore, from the perspective of preventing undernutrition in old age, it is extremely important to assess the mild risk of undernutrition or the risk of future undernutrition in the general older population, and to take appropriate measures at an earlier stage.

There are already a variety of scales for assessing undernutrition in the elderly, such as MNA^®^ (Mini Nutritional Assessment)^8^ and SGA (Subjective Global Assessment),^9^ and these scales have been validated in the older population in Japan.^10^ However, these scales are used mainly to evaluate older persons who require nursing care or are hospitalized and are not suitable for assessing mild risk of undernutrition or the risk of future undernutrition among community-dwelling older adults. On the other hand, the SCREEN (Seniors in the Community: Risk Evaluation for Eating and Nutrition)^11^ and the DETERMINE (DETERMINE Your Nutritional Health Checklist)^12^ tools have been developed in Western countries as screening instruments for low nutrition risk in the community elderly, and attempts have been made to examine the validity in Japanese subjects.^13^^,^^14^ However, the distribution of risk factors for undernutrition, irrespective of host or environmental factors, differs greatly between the Western and Japanese community elderly, so the scales developed in the West cannot be used as they are. Furthermore, no scale has been available so far that can assess the risk of future undernutrition, even if the person is not yet undernourished.

The nutritional status of older persons is closely related to the level of individual physical and mental functions, such as cognitive function,^15^ chewing function,^16^^,^^17^ and walking function,^18^^,^^19^ as well as to the diseases they suffer from. It is also greatly influenced by external and environmental factors that affect nutritional intake, such as family structure (eg, presence of family members living together)^20^^,^^21^ and local food environment (eg, accessibility to groceries).^22^ Furthermore, diet-related literacy and quality of life, which have not been considered in previous scale development, have also been reported to influence dietary intake.^23^^,^^24^ In order to assess the risk of early undernutrition in the general older population, it is necessary to identify important items among these various factors.

In this study, we first developed a preliminary questionnaire, including items covering the four domains that may affect nutritional intake based on previous studies and novel additional items, and introduced it into epidemiological studies of community-dwelling older adults. The data obtained were used to refine the items according to general scale development procedures, and its reliability and criterion-related validity were examined.

The ultimate goal of this study is to develop a simple and self-administered assessment tool to evaluate the risk of mild undernutrition or future undernutrition for community elderly.

## METHODS

### Subjects

The subjects were a total of 1,192 participants; 478 older persons who participated in the Hatoyama Cohort Study 2014 survey (a follow-up survey four years after the 2010 baseline survey) and 714 those who participated in the Kusatsu Town Study 2015 survey (a survey conducted in conjunction with health examinations for the elderly in the same town). Both Hatoyama Cohort Study (2010–present) and Kusatsu Town Study (2001–present) have been conducted by the Tokyo Metropolitan Institute of Gerontology, and their details were described elsewhere.^25^^,^^26^

### Development of candidate items

The position paper of the Academy of Nutrition and Dietetics envisions four domains related to the nutritional status of the elderly: (1) medical and health status, (2) physical and functional status, (3) cognition, and (4) environment.^27^ In order to cover the content included in these domains, a preliminary questionnaire was developed consisting of 40 items from previous studies on the nutritional status of the elderly and 8 items newly added in this study, for a total of 48 items.^28^^–^^31^ The sources of the questions (eg, reference numbers) and what previous research (mainly on the elderly in Japan) has revealed, and what it means to set questions, are also noted in eTable 1.

### Preliminary analysis to select items

Preliminary analysis to select items was conducted in the Hatoyama Cohort Study 2014 survey. Participants were asked to complete a questionnaire consisting of 48 items. The results were used to calculate pass/fail rates and non-response rates, as well as to determine whether there were gender and regional differences (old area/new area of the town) in each item, and to refine the items. As the results, 34 items were selected.

### Analysis to select the final items

In the following year, we undertook the Kusatsu Town Study 2015 survey, in which the 34 items were used, mainly because of reducing the burden on the subjects. However, regarding six items out of 34 items, we either merged the selection categories or changed the wording to something easier to understand. Therefore, 34 items are basically common to both samples and were used in the subsequent analysis.

The 48 items used in the Hatoyama Cohort Study 2014 survey and the 34 items used in the Kusatsu Town Study 2015 survey are shown in Table 1. Both the data of the Hatoyama Cohort Study 2014 and the Kusatsu Town Study 2015 were combined and used for developing the new scale with special reference to previous studies.^32^^,^^33^

**Table 1.** List of candidate items and item selection process

Questions		Total *N* = 1,192 Passage rate (%)	Non- response rate (%)	(48 items)	(34 items)	Non- response rate (%)	Regional differences Effect size ϕ	Male Passage rate (%)	Female Passage rate (%)	Sex difference Effect size ϕ	Discriminability	Factor analysis Commonality (first time)	^c^Reason for Item Deletion
				Hatoyama 2014 *N* = 478 Passage rate (%)	Kusatsu 2015 *N* = 714 Passage rate (%)								
(1)	Have you experienced mental stress or acute illness in the past 3 months?	88.4	0.6	90.4	87.1	0.8	0.042	91.5	85.6	0.083	0.309	0.144	Commonality
(2)	Have you been hospitalized in the past year?	90.5	0.4	91.0	90.2	0.6	0.008	89.0	91.9	−0.063	0.231	0.066	Commonality
(3)	Do you take more than 4 medications a day?			66.9									
(4)	Do you feel that your sense of taste has changed recently?			96.0									Passage rate
(5)	Do you feel your sense of smell has changed in these days?			96.2									Passage rate
(6)	Has the shape of food changed in these days? (For example, to make it easier to eat, make it smaller or softer.)	86.7	0.3	85.4	87.5	0.6	−0.039	89.4	84.1	0.069	0.347	0.229	
(7)	Has the type of food changed in these days?	93.6	0.4	93.9	93.4	0.7	−0.003	94.1	93.2	0.002	0.269	0.148	Commonality
(8)	Do you have dental, oral, or swallowing problems?	83.9	0.3	81.2	85.7	0.6	−0.068	84.1	83.7	−0.001	0.324	0.134	Commonality
(9)	Do you have persistent diarrhea?			97.9									
(10)	Do you have persistent constipation?	89.8	0.3	90.6	89.4	0.6	0.012	91.5	88.3	0.043	0.260	0.069	Commonality
(11)	Compared to people of the same age, do you think your health is good?	77.3	0.8	82.8	73.7	1.0	0.104	80.0	74.9	0.057	0.438	0.171	
(12)	Do you control your diet under the guidance of a physician, nutritionist, or other professional?	88.9	0.4	90.8	87.7	0.7	0.040	89.7	88.2	0.013	0.102		Discriminability
(13)	Do you have difficulty with eating posture or eating movements?			99.0									Passage rate
(14)	Do you feel inconvenienced in preparing meals by yourself (or by your cook)?			96.0									Passage rate Discriminability
^a^(15)	Which of the following applies to your daily mobility? <Able to go out alone by bicycle, car, bus or train → Yes/No in the left column>	95.8	0.5	95.8	95.8	0.6	−0.003	98.4	93.4	0.110	0.168		
(16)	Compared to last year, do you go out less often?	83.5	0.4	85.6	82.1	0.7	0.039	85.7	81.4	0.050	0.419	0.302	
(17)	Do you often trip or slip in the house?	88.8	0.4	91.6	84.3	0.6	0.089	90.6	87.2	0.139	0.321	0.132	Commonality
(18)	Have you lost 3 kg or more in the last 6 months?	87.8	0.4	93.1	87.0	0.6	0.129	88.3	87.4	0.004	0.280	0.086	Commonality
(19)	During the past 6 months, do you think your body has lost more muscle or fat than before?	70.9	0.4	74.9	68.2	0.6	0.070	72.0	69.9	0.016	0.474	0.261	
(20)	Do you feel less physically active on a daily basis?	80.6	0.3	81.6	80.0	0.6	0.015	81.2	80.1	0.007	0.519	0.435	
(21)	Do you feel less motivated to eat?	89.7	0.4	90.4	89.2	0.7	0.009	90.1	89.3	0.001	0.409	0.391	
^a^(22)	Do you enjoy daily mealtime? <2 choices Yes/No>	89.4	0.3	67.8	86.1	0.6	0.125	90.1	88.8	0.011	0.354	0.361	
(23)	Do you try to keep your mood as upbeat as possible?	88.4	0.4	87.7	88.9	0.7	−0.030	86.1	90.6	−0.079	0.207	0.155	
(24)	Are you satisfied with our current dietary habits? <4 choices Yes>			63.6									
^a^(25)	Do you think daily meals are tasty? <2 choices Yes/No>	94.9	0.4	82.2	93.7	0.7	0.081	95.2	94.7	0.005	0.310	0.451	
(26)	Do you eat less than two meals a day?			97.1									Passage rate
^b^(27)	Do you eat less staple foods (eg, rice)?	74.0	0.5	69.0	77.3	0.8	−0.100	72.6	75.2	−0.038	0.409	0.277	
^b^(28)	Do you eat less main dishes (meat, fish or other side dishes)?	82.6	0.4	82.2	82.8	0.7	−0.015	83.4	81.7	0.014	0.443	0.364	
^b^(29)	Do you consume little milk or dairy products?	77.5	0.4	81.0	75.2	0.7	0.062	76.8	78.2	−0.024	0.291	0.074	Commonality
(30)	Do you consume little milk or dairy products?	85.6	0.3	88.9	83.3	0.6	0.072	82.9	88.0	−0.081	0.283	0.111	Commonality
(31)	Do you drink at least 3 glasses of water (eg, water, juice, coffee, tea, milk) per day?			97.9									
(32)	Do you avoid eating meat and eggs for your own health?	92.1	0.5	93.9	90.9	0.8	0.042	93.7	90.6	0.048	0.152		
(33)	Are you interested in nutrition and diet?	87.5	0.4	90.2	85.7	0.7	0.058	83.3	91.4	−0.136	0.320	0.352	
(34)	Do you have the knowledge and skills necessary for proper food selection and meal preparation?			53.1									
(35)	Do you refer to the food labelling when selecting foods and dishes?			32.8									
(36)	Do you take in foods that are said to be good for your health?	81.5	0.8	83.3	80.4	0.9	0.031	74.4	88.2	−0.197	0.356	0.386	
(37)	Do you have any roommates currently living with you (living on the same premised)?	81.3	0.4	91.4	74.5	0.6	0.211	87.8	75.2	0.154	0.288	0.588	
(38)	Do you have financial reasons that prevent you from eating enough food?			98.1									
(39)	How would you rate your household’s current living conditions? <5 choices, 3 Normal to 5 Fairly comfortable>	89.1	0.5	92.9	86.6	0.7	0.095	89.9	88.3	0.011	0.260	0.073	
^b^(40)	Do you often eat alone?	68.3	0.5	72.8	65.3	0.8	0.074	73.0	63.9	0.091	0.344	0.655	
(41)	Do you try to take more opportunities to eat with family or friends?	74.1	0.5	81.0	69.5	0.8	0.123	73.7	74.4	−0.014	0.418	0.314	
(42)	Do you exercise often and try to balance the amount of food you eat?	77.1	0.7	78.7	76.1	0.9	0.024	74.4	79.6	−0.075	0.487	0.416	
(43)	Do you try to lead a regular lifestyle?	90.8	0.4	93.5	88.9	0.7	0.068	84.9	92.6	−0.079	0.354	0.315	
(44)	Are you getting enough rest and sleep?	89.8	0.6	91.8	88.5	0.9	0.041	90.8	89.0	0.018	0.292	0.087	
^b^(45)	Do you feel it is inconvenient for you (or your cook) to go shopping for food?	89.2	0.4	97.1	83.9	0.7	0.202	94.1	84.6	0.143	0.293	0.115	
(46)	Do you ever look forward to eating or making (or helping to make) meals in your relationships with others?			67.6									
(47)	Do you talk about health and nutrition with your family, relatives, friends, and neighbors?	74.7	0.3	75.3	74.4	0.6	0.008	66.4	82.5	−0.193	0.291	0.244	
(48)	Do you try to use your hobbies and interests to make new friends?			64.2									

^a^Changed multiple choices to two choices.

^b^Revised item wording.

^c^Item removal criteria: Pass rate greater than 95%, non-response rate less than 1%, discrimination less than 0.2, factor analysis commonality less than 0.15.

First, item analysis was conducted on the 34 items, and items were selected based on the following criteria: adequate pass rate (the rate at which the item is judged to be risk-free) and discriminative power (the correlation coefficient between each item score and the test score), no gender or regional differences between items, and a certain level of commonality based on factor analysis. Next, after examining the factor structure of the candidate items through exploratory factor analysis, confirmatory factor analysis was conducted as the final scale structure.

### Examination of criterion-related validity of the new index

Criterion-related validity of the new index was examined by determining Spearman’s partial rank correlation coefficients^34^ (adjusted for sex and age) between scores on the developed index and important health indicators of the elderly (higher-order functional capacity, frailty index, mental health, physical function and structure, and cognitive function) and dietary intake status (diversity of food intake).

### Data collection and analysis

In both the Hatoyama and Kusatsu surveys, questionnaires were distributed to the subjects in advance, asking about candidate items related to index creation, sex, age, whether or not the subject lived alone, medical history, the TMIG-Index of Competence,^35^ the frailty index (Kaigo-Yobo Checklist^36^ [Hatoyama] or Kihon Checklist^37^ [Kusatsu]), the Geriatric Depression Scale 15 items short version (GDS-15),^38^ and Dietary Variety Score (DVS).^39^

Briefly, the TMIG-Index of Competence is a scale that measures functional capacity higher than basic activities of daily living; 13 questions consisting of four subscales (eg, can you do the following) are answered with a score of 1 for “yes” and 0 for “no,” and the score is calculated (full total score of 13). The higher the score, the higher the functional capacity. Similarly, the DVS is a tool to assess dietary variety with a food intake frequency questionnaire covering the 10 major foods in the Japanese diet (maximum score of 10). Higher scores indicate greater dietary variety.

The self-administered survey form was brought on the day of the medical checkup, and the investigator checked the completed form. Mini-Mental State Examination (MMSE)^40^ was conducted by interview by an investigator who had received training for standardization and accuracy control of the survey in advance. Grip strength, usual walking speed, and time to stand on one leg with eyes open (up to 60 sec) were also measured as usual at the site of the medical checkup by examiners who had received training for standardization and accuracy control of the tests in advance. Body composition was measured with the body component analyzer InBody730 (InBody Co., Tokyo, Japan), and skeletal mass index (SMI) was calculated from the results.

All statistical analyses in this study were performed using IBM^®^ SPSS Statistics 27 (IBM Corp, Armonk, NY, USA). The statistical significance level was set at less than 5%.

### Ethical considerations

Participants were informed of the purpose of the study, the handling of personal information, and data usage for research purpose only, and signed the consent forms. The Hatoyama Cohort Study and the Kusatsu Town Study were approved by the Ethics Committee of the Tokyo Metropolitan Institute of Gerontology (or the Ethics Committee of the Research Division of the Tokyo Metropolitan Geriatric Hospital and Gerontology Center) (Kusatsu Town Study, August 13, 2003: 15 Zai Kenkyu No. 870; Hatoyama Cohort Study, August 5, 2010: receipt number 32), and additional approvals were obtained from the same committee as needed thereafter.

## RESULTS

### Basic attributes of the participants

The basic attributes of the participants analyzed are shown in Table 2. By gender, 574 (48.2%) were male and 618 (51.85%) were female, with an overall mean age of 74.7 (standard deviation, 5.8) years (range, 65–93 years). In terms of medical history, 46.6% had hypertension, 28.5% had dyslipidemia, 13.0% had diabetes mellitus, 11.6% had cancer, 8.2% had heart disease (excluding arrhythmia), and 5.8% had stroke.

**Table 2.** Basic attributes of the participants

Indicators	Attribute	Total number *N* = 1,192	Hatoyama 2014 *N* = 478	Kusatsu 2015 *N* = 714
Sex	Male, *n* (%)	574 (48.2%)	288 (60.3%)	286 (40.5%)
	Female, *n* (%)	618 (51.8%)	190 (39.7%)	428 (59.9%)
Age	Years, Mean (SD) [range]	74.7 (5.8) [65–93]	75.1 (4.7) [69–89]	74.4 (6.4) [65–93]
Family structure	Living alone, *n* (%)	219 (18.4%)	41 (8.2%)	178 (24.9%)
Medical history	Hypertension, *n* (%)	555 (46.6%)	232 (46.5%)	323 (45.2%)
	Diabetes Mellitus, *n* (%)	155 (13.0%)	68 (14.2%)	87 (12.2%)
	Cancer, *n* (%)	138 (11.6%)	60 (12.6%)	78 (10.9%)
	Stroke			27 (3.8%)
	Cerebral infarction, *n* (%)	54 (4.5%)	27 (5.6%)	4 (0.6%)
	Cerebral hemorrhage, *n* (%)	8 (0.7%)	4 (0.8%)	5 (0.7%)
	Subarachnoid hemorrhage, *n* (%)	7 (0.6%)	2 (0.4%)	14 (2.0%)
	Heart disease			13 (1.8%)
	Angina pectoris, *n* (%)	33 (2.8%)	19 (4.0%)	43 (6.0%)
	Myocardial infarction, *n* (%)	26 (2.2%)	13 (2.7%)	12 (1.7%)
	Arrhythmia, *n* (%)	100 (8.4%)	57 (11.9%)	212 (29.7%)
	Others, *n* (%)	38 (3.2%)	26 (5.4%)	
	Dyslipidemia, *n* (%)	340 (28.5%)	128 (26.8%)	

SD, standard deviation.

### Selection process of candidate items

Of the 34 candidate items, only one item (Number 15) had a pass rate of 95% or higher. In addition, three items (Numbers 12, 15, and 32) had a discrimination power (I-T correlation coefficient) of less than 0.2, and we decided to exclude these three items.

Next, the pass rates for each item were calculated by gender and region, and significant differences were confirmed by the chi-square test. In addition, φ coefficients were calculated to examine the effect size. Significant differences were found for 14 of the 34 items by gender and for 17 items by region. Of these, none of the items corresponded to φ > 0.3, which is a moderate effect size based on Cohen’s criteria.

Based on these results, 31 items were selected that met the following three criteria: (1) adequate passage rate (<95%), (2) adequate discriminative power (>0.2), and (3) φ coefficient on passage rate by gender and region was less than 0.3.

### Examination of factor structure

**Figure 1.**
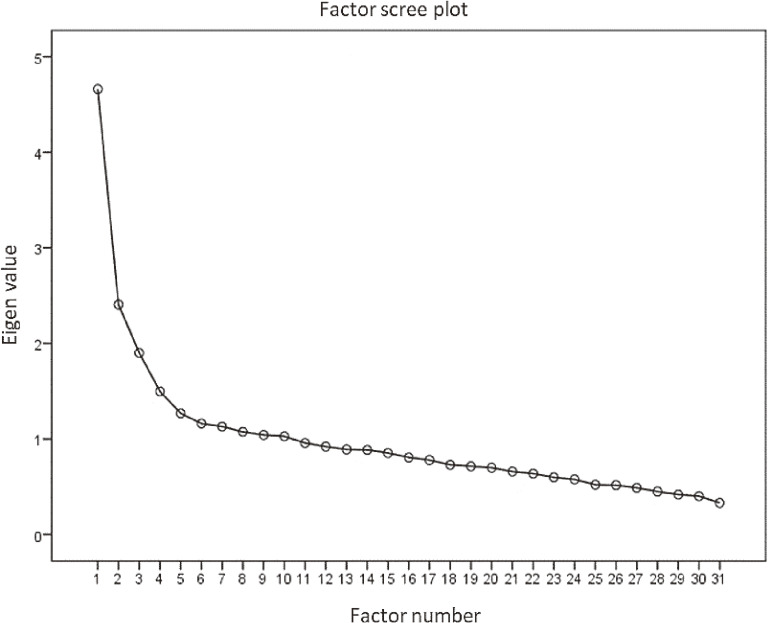
Factor scree plot of candidate items (31 items). A four-factor solution was considered appropriate.

Exploratory factor analysis (repeated main factor method, promax rotation) was conducted on the 31 items. From the scree plot, a four-factor solution was considered appropriate (Figure 1). Next, the number of factors was fixed at four, and factor analysis was conducted again using the main factor method. The analysis was repeated, deleting items with low commonality, and finally a semantic cohesion of 13 items was obtained (Table 3).

**Table 3.** Factor structure of the new index items based on exploratory factor analysis

Overall 13 items (Cronbach’s α = 0.76)	1	2	3	4
Factor 1: Health beliefs (Cronbach’s α = 0.71)				
(36) Do you take in foods that are said to be good for your health?	0.73	0.05	−0.06	−0.09
(33) Are you interested in nutrition and diet?	0.64	−0.01	−0.03	−0.01
(47) Do you talk about health and nutrition with your family, relatives, friends, or neighbors?	0.52	0.04	−0.01	−0.04
(43) Do you try to lead a regular lifestyle?	0.47	−0.06	−0.02	0.14
(42) Do you exercise often and try to balance the amount of food you eat?	0.46	−0.03	0.23	0.11
Factor 2: Dietary status (Cronbach’s α = 0.71)				
(27) Do you eat less staple food (eg, rice)?	0.00	0.78	−0.04	−0.09
(28) Do you eat less main dishes (meat, fish, or other side dishes)?	0.04	0.69	0.02	−0.01
(21) Do you feel less motivated to eat?	−0.03	0.46	0.11	0.24
Factor 3: Physical activity (Cronbach’s α = 0.62)				
(20) Do you feel less physically active on a daily basis?	−0.04	−0.04	0.89	−0.03
(16) Compared to last year, do you go out less often?	0.04	0.00	0.58	−0.07
(19) During the past 6 months, do you think your body lost more muscle and fat than before?	−0.05	0.11	0.38	0.02
Factor 4: Food-related quality of life (Cronbach’s α = 0.71)				
(25) Do you think daily meals are tasty?	−0.01	0.02	−0.05	0.78
(22) Do you enjoy daily mealtime?	0.02	−0.05	−0.02	0.74
Factor contribution rate	21.86	9.30	6.52	5.12
Correlation between factors	1	0.19	0.38	0.37
	2	—	0.41	0.38
	3		—	0.37

Factor extraction method: Repeated main factor method, Promax rotation.

Cumulative contribution rate: 42.80%.

Factor 1 was named “health beliefs” because of the high loadings of items such as consumption of healthy foods, interest in nutrition and diet, and talking about health and nutrition-related matters. Factor 2 was named “dietary status” because it covered items about decreased intake of foods such as staple foods and main dishes, and willingness to eat. Factor 3 was named “physical activity” because it had high factor loadings on items related to physical inactivity, decreased frequency of going out, and decreased muscle and fat mass. Factor 4 was named “diet-related quality of life” due to high factor loadings on items related to meal taste and enjoyment.

**Figure 2.**
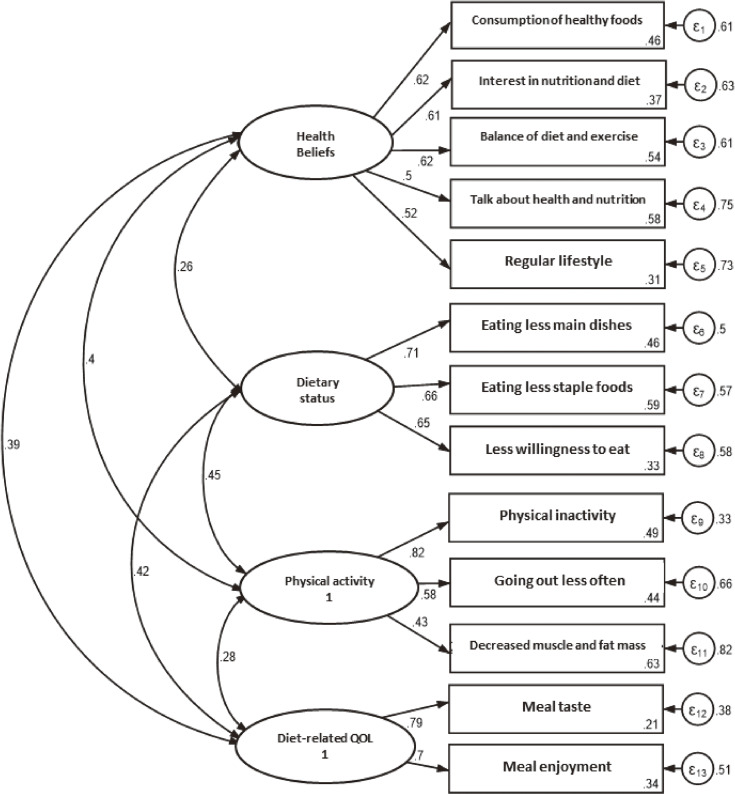
Factor structure of the new index items based on confirmatory factor analysis. The parameters concerning model fitness are shown in the figures.

A confirmatory factor analysis of the four-factor solution was conducted for the 13 selected items (Figure 2). The results showed good model fit, χ^2^(59) = 275.4 (*P* < 0.001), comparative fit index of 0.930, and root mean square error of approximation of 0.056. The range of factor loadings for each factor was 0.43–0.82. If the absolute factor loadings were greater than 0.50, the factors were considered to have sufficient common factors.^41^ Sufficient loadings were indicated for 12 of the 13 items, which had factor loadings of 0.50 or greater. Therefore, we called the index consisting of these four factors and 13 items the “new index” and examined its reliability and validity.

### Reliability and criterion-related validity

**Figure 3.**
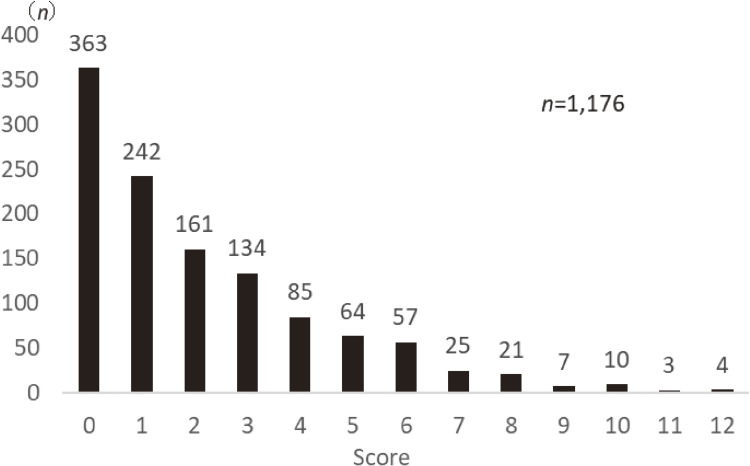
Score distribution of the new index among all participants.

The distribution of scores on the new index was calculated for the 1,176 subjects for whom scores could be calculated (Figure 3). About half of the respondents (51.4%; *n* = 605) scored 0 or 1. In a Good-Poor analysis, the mean scores for each item were compared between groups using a general linear model adjusted for sex and age, with those scoring 1 or less divided into the low-scoring group and those scoring 2 or more into the high-scoring group (Table 4). The mean scores of the high-scoring group were statistically significantly higher for all items (all *P* < 0.001). Reliability coefficients (Cronbach’s α) were calculated using the internal consistency method, resulting in α = 0.76 for all 13 items, α = 0.71 for factor 1, α = 0.71 for factor 2, α = 0.62 for factor 3, and α = 0.71 for factor 4 (Table 3).

**Table 4.** Mean scores of the new index items in Good-Poor analysis

Item	Low scoring group (≦1 point) *N* = 605	High scoring group (≧2 points) *N* = 571	*P*-value
(36) Do you take in foods that are said to be good for your health?	0.03	0.34	<0.001
(33) Are you interested in nutrition and diet?	0.01	0.24	<0.001
(47) Do you talk about health and nutrition with your family, relatives, friends, and neighbors?	0.04	0.42	<0.001
(43) Do you try to lead a regular lifestyle?	0.07	0.44	<0.001
(42) Do you exercise often and try to balance the amount of food you eat?	0.01	0.18	<0.001
(28) Do you eat less main dishes (meat, fish, or other side dishes)?	0.02	0.33	<0.001
(27) Do you eat less staple foods (eg, rice)?	0.08	0.43	<0.001
(21) Do you feel less motivated to eat?	0.00	0.21	<0.001
(20) Do you feel less physically active on a daily basis?	0.01	0.38	<0.001
(16) Compared to last year, do you go out less often?	0.02	0.36	<0.001
(19) During the past 6 months, do you think your body has lost more muscle or fat than before?	0.10	0.48	<0.001
(25) Do you think daily meals are tasty?	0.00	0.09	<0.001
(22) Do you enjoy daily mealtime?	0.02	0.19	<0.001

Subjects were 1,176 persons who responded to all items.

*P*-values are based on a general linear model adjusted for sex and age.

Next, we examined the criterion-related validity of the new index, using important health indicators and dietary intake status of older people as external criteria. Spearman’s partial rank correlation coefficients with sex and age as adjustment factors are shown in Table 5. The GDS-15 and the Frailty Index (Kaigo-Yobo Checklist or Kihon Checklist) had correlation coefficients of 0.5 or higher. Next were higher-order functional capacity (TMIG-Index of Competence) (correlation coefficient of 0.3 or higher) and DVS (0.3). Although the correlation coefficient was less than 0.3, statistically significant associations were also found with cognitive function (MMSE) and the four physical functions and structures (all *P* < 0.001).

**Table 5.** Criterion-related validity of the new index

Health Indicator		^*^Partial Correlation Coefficient	*P*-value
Higher-order functional capacity			
TMIG-IC	Total score	−0.326	<0.001
	Instrumental ADL subscale score	−0.102	<0.01
	Intellectual Activity subscale score	−0.311	<0.001
	Social Role subscale score	−0.277	<0.001
Frailty index			
Kaigo-Yobo Checklist score (Hatoyama only)		0.542	<0.001
Kihon Checklist, 20-item score (Kusatsu only)		0.513	<0.001
Mental health			
GDS-15 score		0.564	<0.001
Cognitive function			
MMSE score		−0.166	<0.001
Dietary intake			
DVS score		−0.298	<0.001
Physical function and structure			
Grip strength		−0.182	<0.001
Usual walking speed		−0.218	<0.001
One leg standing time with eyes open		−0.252	<0.001
Skeletal muscle index		−0.117	<0.001

ADL, activities of daily living; DVS, Dietary Variety Score; GDS-15, Geriatric depression scale 15-items short version; MMSE, Mini-Mental State Examination; TMIG-IC, Tokyo Metropolitan Institute of Gerontology-Index of Competence.

^*^Spearman’s partial rank correlation coefficients adjusted for sex and age.

## DISCUSSION

In this study, a preliminary questionnaire consisting of 48 questions that were thought to influence the food intake and nutritional status of older people was developed, from which a “new index” consisting of 13 items with 4 factors was created through a rigorous scale development process. Four factors were named “health beliefs” (5 items), “dietary status” (3 items), “physical activity” (3 items), and “diet-related quality of life” (2 items). Although the number of items was smaller than the 48 items in the preliminary questionnaire, the reliability coefficients (Cronbach’s α) based on the internal reliability of the 13 items as a whole and the items comprising each factor were high, and the model fit of the four 13-item factors and the factor loadings for each factor were also at satisfactory levels. Furthermore, the index was shown to have adequate criterion-related validity when used as a criterion for important health indicators in old age and food intake diversity.

The inclusion of items related to “health beliefs” and “diet-related quality of life” in this index is a feature not found in other indices for assessing low nutrition risk. SCREEN^11^ and DETERMINE,^12^ screening instruments for low-nutrition risk among the community elderly, do not include items related to these two factors. On the other hand, items related to dietary status and physical activity are included, both of which are characterized by reduced dietary intake and physical activity. Reduced dietary intake and physical activity are considered important items for assessing the risk of low nutrition in the general older population, both domestically and internationally.^42^ In this connection, the MNA^®^^8^ and SGA,^9^ which are indices for assessing low nutrition in hospitalized patients and those requiring nursing care, include low BMI and impaired mobility as endpoints. The decreased dietary intake and physical activity seen in the general older population is thought to lead to low BMI and mobility impairments as the functional decline becomes more severe.^43^ Thus, it is important to identify mild decline in dietary intake and physical activity among the elderly in the community at an early stage, and to take appropriate measures to prevent subsequent decline in functioning.

Compared to the two factors of “dietary status” and “physical activity,” the two factors of “health beliefs” and “diet-related quality of life” are higher-order life functions. First, “health beliefs” is a factor consisting of items related to healthy living and eating behaviors, such as “I try to lead a regular life,” “I am physically active and keep a balance with the amount of food I eat,” and “I take in foods that are said to be good for health”. Thus, the “health beliefs” factor can be described as health literacy. In general, higher health literacy is associated with better subsequent health status and longer life expectancy as the ultimate outcome. In a classic study, Breslow et al examined the association between the practice of seven health habits and subsequent life expectancy and found that the greater the number of health habits practiced, the longer the life expectancy.^44^^,^^45^ The “health beliefs” factor in this study may lead to the prevention of later decline not only in nutritional state, but also in overall life function.

The “diet-related quality of life” factor consists of two items related to the taste and enjoyment of diet. These two items correspond to “enjoyment of meals” and “satisfaction with meals,” two of the four factors that make up the 18-item “Diet-Related QOL Scale” for older people in Japanese communities. Iwasa et al reported that the “Diet-Related QOL Scale” correlated with a wide range of health-related indicators, including subjective well-being, food satisfaction, appetite, food information gathering, and diversity of food intake.^23^ In addition, Takemi et al point out that among the diet-related QOL, quality improvement in eating habits, such as enjoying meal and being interested in meal, plays an important role in improving overall quality of life.^28^ The new “diet-related QOL” factor is expected to help prevent not only low nutrition later on, but also decline in overall life function.

The new index was confirmed to have criterion-related validity for important health indicators in old age, including daily functioning. In particular, it was strongly associated with the GDS-15 and the frailty index, and also moderately associated with higher-order functional capacity (the TMIG-Index of Competence) and diversity of food intake (DVS). Low nutrition in old age is not a problem in itself, but rather a problem in that low nutrition can lead to a decline in the functions of various organs, which in turn can easily lead to impaired functional health.^42^ The fact that this index was found to have a strong association with the daily function and frailty indices suggests the potential significance of this index; namely, the possibility of predicting not only the future risk of low nutrition, but also the risk of future decline in functioning, such as frailty.

On the other hand, the new index showed significant but minor association with objectively measured physical and mental functions. The reasons why the strength of the associations varied depending on health indicators included the similarity of some of the questions in the new index and the external criteria (eg, frailty index), the possible influence of the external criteria on the scores of the new index (eg, GDS-15), and the possibility that the risk of early undernutrition itself may still have only a minor effect on objectively-measured physical and mental function and structure (eg, physical strength and function and MMSE). Further investigation is required to address this point.

Finally, we must point out an important limitation of this study: the representativeness of the sample. First, the two samples may not be representative of the general older population in their respective areas. The Hatoyama Cohort 2014 study participants were followed up 4 years after the Hatoyama cohort started in 2010, and during this period dropouts occurred for reasons such as death, hospitalization, and relocation. The Kusatsu Town Study 2015 was also a research study conducted on participants in the annual health checkups conducted by the town. In general, the health checkup participants are, on average, a relatively healthy group with a bias. Second, the two areas from which the sample was drawn were local municipalities in Saitama and Gunma Prefectures, which are in the Kanto area in Japan. There are regional disparities in the health and food environment of the elderly that cannot be ignored, even within Japan. The generalization of the new indicators created in this study needs to be verified in the future.

Nevertheless, the new index created in this study consists of 13 items and four factors that represent “health beliefs,” “dietary status,” “physical activity” and “diet-related quality of life”. As discussed above, these factors have plausibility as predictors of early undernutrition risk or future occurrence of undernutrition in the general older population, suggesting the high applicability in other regions. In the near future, we would like to conduct follow-up studies to examine the predictive validity of this index and to seek a cutoff point for screening high-risk individuals.

## SUPPLEMENTARY MATERIAL

eTable 1. 48 questionnaire items and sources, and meaning of question setting
