# Supplementary material for: Development of a Brief Assessment Tool to Evaluate Early Low Nutrition Risk in Community Elderly: Creation of the Tool and Examination of Its Reliability and Criterion-related Validity
Source: J Epidemiol. 2025 Feb 5;35(2):71–80. doi: 10.2188/jea.JE20240056 (PMC11706674; doi:10.2188/jea.JE20240056)
Supplement: Supplementary file 1 [file je-35-071-s001.pdf]

eTable 1. 48 questionnaire items and sources, and meaning of question setting

Major items	Medium items	no.[a]	Question	Source (reference number [b])	Hatoyama Cohort Study 2014 survey [c] items	What previous research (mainly on the elderly in Japan) has revealed, and what it means to set questions.
Medical/ Health Status	Presence of chronic or acute illnesses	1	Have you experienced mental stress or acute illness in the past 3 months?	30		
		2	Have you been hospitalized in the past year?	29	Questionnaire distributed in advance Q3	Physiological stress may cause a decrease in serum albumin levels.
	Medication use	3	Do you take more than 4 medications a day?	29,30,31		>5 kinds in the assessment for prevention of low nutritional status, >4 kinds in the MNA, and >3 kinds in the Nerima Ward questionnaire. It has been noted that if a person is regularly taking multiple types of medications, it is likely that their nutritional status is also poor.
	Sensory changes - taste, smell, appearance, texture	4	Do you feel that your sense of taste has changed recently?	Newly created		2014 Kusatsu survey, lifestyle questionnaire item (introduced at the request of dentists) “How is the taste of your food compared to when you were 50?" Five choices: very bad, bad, about the same, good, and very good.
		5	Do you feel your sense of smell has changed in these days?	Newly created		
		6	Has the shape of food changed in these days? (For example, to make it easier to eat, make it smaller or softer.)	Newly created		
		7	Has the type of food changed in these days?	Newly created		
	Oral health	8	Do you have dental, oral or swallowing problems?	29		Insufficient food intake, possibly secondary to PEM.
	Others	9	Do you have persistent diarrhea?	29		"Do you have persistent diarrhea or use laxatives regularly?" Possible loss of fluids and nutrients. The same applies to regular use of laxatives for constipation.
		10	Do you have persistent constipation?	29		Possible loss of gastrointestinal tract function, resulting in decreased digestion and absorption efficiency.
		11	Compared to people of the same age, do you think your health is good?	30		MNA question, "Compared to others your age, how do you feel about your health?" Four choices: not good, not sure, same, good."
		12	Do you control your diet under the guidance of a	Newly		

			physician, nutritionist or professional?	created		
Physical/ Functional status	Physical limitations	13	Do you have difficulty with eating poster or eating movements?	29		Inappropriate eating posture and eating movements may result in decreased appetite and inadequate intake.
		14	Do you feel inconvenienced in preparing meals by yourself (or by your cook)?	29		External factors, such as difficulty preparing meals, may lead to poor nutritional status, even if appetite is good.
		15	Which of the following applies to your daily mobility? <Able to go out alone by bicycle, car, bus or train → Yes/No in the left column>		Questionnaire distributed in advance Q4	
		16	Compared to last year, are you going out less often?	31		People who are less likely to go out or are not out at least once a week may not be able to take in a variety of foods, are not getting the nutrients they need from their diet, or are in poor health.
	Balance	17	Do you often trip or slip in the house?		Questionnaire distributed in advance Q11	
	Physical strength and endurance	18	Have you lost 3 kg or more in the last 6 months?	29,31	Questionnaire distributed in advance Q8	They may have decreased energy intake, decreased efficiency of digestion and absorption, or increased energy consumption. Energy consumption may be updated by disease; 6 months is a rough estimate.
		19	Do you think you have lost more muscle and fat from your body in the last 6 months than before?	29	Questionnaire distributed in advance Q9	Decreased protein and energy intake and decreased physical activity may be present. 6 months is a rough estimate.
	Physical activity	20	Are you no longer physically active on a daily basis?	29		Decrease in daily physical activity may be due to decreased food intake due to decreased appetite rather than excess energy in the elderly.
Cognition	Changes in mental status	21	Do you feel less motivated to eat?<br><2 choices Yes/No>	29		"Have you lost the energy to eat?"<br>Decreased appetite not only decreases food intake, but also affects the autonomic nervous system and hormones, resulting in decreased food or absorption efficiency.
	Depression	22	Do you enjoy you daily mealtime?	29	Questionnaire on the day of the survey Q19	When depressive states are observed, the impact on nutritional intake should also be considered
		23	Do you try to keep your mood as upbeat as possible?	31		
	Emotional needs	24	Are you satisfied with our current dietary habits?<br><4 choices Yes>	28	Questionnaire on the day of the survey	"People who are more satisfied with their diet tend to have a more positive outlook on life, such as "the food is delicious" and "the atmosphere at the
		25	Do you enjoy a good meal every day?<br><2 choices Yes/No>	28		

					Q16, 17	dining table is cheerful."<br>... Cabinet Office: 2010 White Paper on Dietary Education (2010)."
	Habitual food intake	26	Do you eat less than two meals a day?	29,30		Less frequent meals may decrease intake and lead to PEM; the MNA asked respondents to choose "once, twice, or three times a day" in the "How many meals do you eat per day?" option.
		27	Are you eating less staple foods (e.g., rice)?	29		Decreased staple food intake can lead to energy and protein deficiencies. Note that when the intake is only 80% or less for a week in a row, it is easy to fall into PEM.
		28	Are you eating fewer mail dishes (meat, fish or other side dishes)?	29,30		Decreased intake of staple foods can cause protein and fat deficiencies. Note that when only 80% or less is consumed for a week in a row, it is easy to fall into PEM. The MNA asks questions in the form of "Do you consume meat or fish daily?" and "Do you consume at least two legumes or eggs weekly?" and scores them on protein intake, along with dairy products.
		29	Do you consume little milk or dairy products?	29,30		Milk and dairy products are a good source of protein, so lack of intake can cause PEM. In the MNA, the question is "Do you consume at least one dairy product (milk, cheese, yogurt) daily?
		30	Do you consume little milk or dairy products?	30		Lower intake of side dishes can cause micronutrient deficiencies.
		31	Do you drink at least 3 glasses of water (water, juice, coffee, tea, milk, etc.) per day?	30		Assessing fluid intake; the MNA question asked, "How much fluid (water, juice, coffee, tea, milk, etc.) do you drink per day?"<br>Three choices: less than 3 glasses, between 3 and 5 glasses, and more than 5 glasses.
	Health/ nutrition-related beliefs	32	Do you avoid eating meat and eggs for your own health?	Newly created		
		33	Are you interested in nutrition and diet?	31		Those who are not interested in nutrition and diet are more likely to be underweight than overweight and do not get enough nutrition from their diet. They also tend to report a less-than-ideal sense of health.<br>Miki Narita: Care Prevention Case Study No. 7: Approaches to People Losing Interest in Diet, Monthly Long-Term Care Insurance, 2010; 176: 22-23."
		34	Do you have the knowledge and skills necessary	31		

			for proper food selection and meal preparation?			
	Advertising	35	Do you refer to the food labelling when selecting foods and dishes?	Newly created		Modified item from the National Health and Nutrition Survey.
		36	Do you incorporate foods that are known to be good for your health?	Newly created		
Environmental	Living situation	37	Do you have any roommates currently living with you (living on the same premised)?		Questionnaire distributed in advance Q37	Question asking whether or not the person lives alone. Having a partner to eat with tends to result in a greater variety of foods being consumed (higher food intake diversity scores). (Presented at a public gerontology meeting by Narita M.).
	Economics	38	Do you have financial reasons that prevent you from eating enough food?	29		Even in the absence of extreme poverty, food intake may decline because of economic status
		39	How would you rate your household's current living conditions? <5 choices, 3 Normal to 5 Fairly comfortable>		Questionnaire distributed in advance Q34	
	Environment	40	Do you often eat alone?	29,31		Those who eat alone are said to have the potential to lower their food intake without realizing it. Eating alone tends to make eating less tasty and enjoyable. Note that solitary eating is more common among women, underweight persons, and those who live alone.
	Lifestyle	41	Do you try to take more opportunities to eat with family or friends?	28		The original text reads: 'Eat with family or friends.' Four choices: most positive, somewhat positive, somewhat negative, and most negative. In addition to family members, those who eat together with separated children, their families, relatives, and friends more frequently and interact with them more frequently through food have better dietary and health status. ・Takemi Y. Relationship between meal sharing status and positive eating behavior and attitude among the elderly who live alone, Ethnic Health, 1997; 63(2): 90-110.
		42	Do you move around a lot and try to balance the amount of food you eat?	31		Decline in activities of daily living not only promotes physical functional decline, such as decreased energy metabolism and skeletal muscle weakness, but also makes people more susceptible to poor nutrition due to decreased appetite, etc. Over a 2-year period, more people in the late elderly became less physically active and less able to eat and balance their diet than before.
		43	Do you try to lead a regular lifestyle?	31		
		44	Are you getting enough rest and sleep?	31		

	Access to food and food preparation	45	Do you feel it is inconvenient for you (or your cook) to go shopping for food?	29		External factors, such as difficulty in going out for shopping, even if one has a good appetite, can lead to a decline in nutritional status.
	Socialization	46	Do you ever look forward to eating or making (or helping to make) meals in your relationships with others?	28		There is a causal effect of better social status on maintaining physical health 3 years later.<br>· Liu X, Hoshi T, et al. Changes over time and their causal effects on physical health and social health among urban homebound elderly, Journal of Health Education, 2008; 16:176-185."<br><br>Getting out of the house, extending and interacting with society and others, and enjoying meals with friends and family are said to promote appetite. However, during the 2-year follow-up period, there was a change in the group of non-specific elderly candidates toward not making new friends, using hobbies and pleasures more than before.
		47	Do you talk to your family, relatives, friends, neighbors, etc. about health and nutrition?	28		
		48	Do you try to use your hobbies and interests to make new friends?	31		

MNA, Mini Nutritional Assessment; PEM, Protein Energy Malnutrition.

[a], "no." corresponds to the question number in Table 1 in the main text.

[b], Reference number corresponds to that in the main text.

[c], Hatoyama Cohort Study 2014 survey included the questionnaire distributed in advance and questionnaire on the day of the survey.
